# Risk Factors and Prediction Model for Non-curative Resection of Early Gastric Cancer With Endoscopic Resection and the Evaluation

**DOI:** 10.3389/fmed.2021.637875

**Published:** 2021-05-14

**Authors:** Xiaoqian Ma, Qian Zhang, Shengtao Zhu, Shutian Zhang, Xiujing Sun

**Affiliations:** Department of Gastroenterology and Hepatology, Beijing Friendship Hospital, Capital Medical University, National Clinical Research Center for Digestive Disease, Beijing Digestive Disease Center, Beijing Key Laboratory for Precancerous Lesion of Digestive Disease, Beijing, China

**Keywords:** early gastric cancer, endoscopy, non-curative resection, ESD, EMR, predictive model

## Abstract

**Background and Study Aim:** EGC, also known as Early Gastric Cancer is known to lack the lymph node metastasis and confined along the mucosa, which is treated through an endoscopic resection procedure that includes ESD (Endoscopic Submucosal dissection) and EMR (Endoscopic Mucosal Resection). However, some cases underwent residual disease, recurrence, or additional gastrectomy because of non-curative resection. The following research aims to delineate the threat factors causing the non-curative resection as well as develop a predictive model.

**Patient and Methods:** Effort was taken to collect all the records about the health history of pathologically diagnosed EGC who experienced endoscopic treatment in the Department of Endoscopy, the Capital Medical University, and Beijing Friendship Hospital from January 2012 to January 2020. Patients were grouped into two categories primarily; a curative resection group and finally a non-curative resection group based on the outcomes of the postoperative pathological and immunohistochemical examination results. The statistical methods used included single factor analysis, a multivariate logistic regression analysis and a chi-square test. A nomogram for the prediction of non-curative resection was constructed, which included information on age, gender, resection method, postoperative pathology, tumor size, ulcer, treatment, and infiltration depth. Receiver operating characteristic (ROC) curve analysis and calibration were performed to present the predictive accuracy of the nomogram.

**Results:** Of 443 patients with 478 lesions who had undergone ESD or EMR for EGCs, 127 were identified as being treated non-curative resection. Older patients (>60 years), a large tumor size (>30 mm), submucosal lesion, piecemeal resection, EMR for treatment and undifferentiated tumor histology were associated with non-curative resection group. Our risk nomogram showed good discriminated performance in internal validation (bootstrap-corrected area under the receiver-operating characteristic curve, 0.881; *P* < 0.001).

**Conclusions:** A validated prediction model was developed to identify people who were subject to undergoing a non-curative resection for ESD. The predictive model that we formulated is essential in providing reliable information to guide the decision-making process on the treatment for EGC before undertaking an endoscopic resection.

## Introduction

EGC, commonly known as Early Gastric Cancer, is the kind of tumorous tissue that affects the gastric submucosa or mucosa in the presence and or absenteeism of lymph node metastasis. Premature detection of EGC can be cured following extensive endoscopy with a 5-year survival rate exceeding 90% (1). Endoscopic treatment refers to an endoscopic resection that includes ESD and EMR (2). Extensive multicenter studies have shown that relative to surgical gastrectomy, the procedures for endoscopic resection entails numerous advantages of less trauma, fewer complications, and high quality of life and it is now widely accepted, particularly in high incidence in Asian countries (3). It has become the first choice for patients with high-grade intraepithelial neoplasia and EGC (4, 5). Suppose endoscopic treatment wants to achieve the same therapeutic effect as surgery. In that case, the prerequisite is that the early lesions must be removed entirely at one time, and the lesions have no threat of lymph node metastasis, which is to achieve the standard for potentially curative resection. Tentatively, a non-curative resection is simply a standardized pathological evaluation of the lesions after resection cannot reach the standard of curative resection. Secondary endoscopic treatment or even surgical treatment is required once there is an occurrence of non-curative resection.

The rates associated with incomplete resection are between 24.6 and 39.5% (6–8). Research reveals that numerous reasons exist behind non-curative resection. The reasons include the failure to undertake en bloc resection because of a preliminary mis-diagnosis of the lesion's penetration and poor technique. ESD is regarded as the best procedure in the cure for early gastric neoplasms. It must, however, be noted that it demands advanced skills in endoscopy, but it does carry with its heightened levels of problems, that include excessive bleeding and increased perforations when equated to routine EMR procedures (9, 10).

Additionally, it is difficult to undertake an ESD for complications associated with lesions of considerable sizes that occur in specific locations. Hence, in a variety of situations, en bloc resection is not appropriate. The forecast for neoplasm depths or margins can be challenging because gastric mucosa's background is affected by chronic and acute inflammation (11). Hence, this can lead to inaccurate prognosis on the depth or margin of the lesions, regardless of the utilization of chromoendoscopy with the indigo carmine dye or the magnification of endoscopy using NBI (Narrow Band Imaging) (11, 12).

Whether it is residual disease or recurrence, secondary endoscopic resection or surgeries have the potential of manifesting into problems for both endoscopists and the patients leading to inflated health care expenses. When taking into account the amount of EMR(s) or ESD(s) performed, including the public desire for reduced invasive medical measures, it is crucial to demystify the person's potential for non-curative resection.

Hence, this research assessed the potential risk factors of non-curable resection in patients suffering from EGC and formulated a predictive model to provide a reference for the prevention and clinical evaluation.

## Experimental Section

### Patients and Methods

We constructively examined clinical data for patients that had undergone endoscopic resection from the Department of Endoscopy, the Capital Medical University affiliated Beijing Friendship Hospital from January 2012 to January 2020.

The characteristics ingrained in the clinicopathology included the sex and age of the patient; their smoking tendency; *Helicobacter pylori* infection; the magnitude and position of the lesions; the histology of the cancer; and the different endoscopic findings of early gastric cancer that include remarkable redness, central depression, interruption or smooth tapering of fold, white fur, and nodularity.

Approval for this project was obtained from the Beijing Friendship Hospital. This research's reporting adapts to the STROBE (Strengthening the Reporting of Observational Studies Epidemiology) guidelines concerning the wider Enhancing the Quality and Transparency of Health Research guidelines.

### Endoscopic Submucosal Dissection Technique

All the ESD measures were conducted on the patients that were hospitalized. Propofol or Midazolam hydrochloride was intravenously administered for sedation purposes prior to the surgery. The affected people were positioned in a left lateral decubitus position and were observed using a typical single-channel endoscope of (GIF-H260Z or GIF-Q260J; from Olympus Optical Co., Ltd., Tokyo, Japan). Following the summation of the endoscopic evaluation for gastric lacerations, care was taken to mark all areas surrounding the lesions with electrocautery (VIO 300D; ERBE, from Tübingen, Germany) by means of the needle knife (Olympus, Tokyo, Japan). To raise the lacerations above the muscle tissue, care is taken to administer a saline rich solution containing high concentration of epinephrine (0.01 mg/mL), including 0.8% of indigo carmine that was later inserted into the patient's submucosal layer using a 21-gauge syringe.

A circumferential dissection and incision were done using a needle knife, including a cloistered tip- knife (KD-610L, from Olympus Optical Co., Ltd.). The vessels that were exposed or bleeding were mitigated using hemostatic forceps or hem clips.

Drugs known to heightened bleeding such as warfarin, non-steroidal anti-inflammatory drugs, and aspirin were withdrawn from 5 to 7 days prior to the endoscopic resection. The drugs mentioned were later restarted 2 weeks after completing EMR or ESD if and when the postoperative bleeding had not developed. The patients were managed using proton pump inhibitors for between 4 and 8 weeks following EMR/ESD.

### Gross and Histopathologic Cross-Examination

The outcomes arrived from endoscopy of EGC were divided along on the standards of Japan's Gastric Cancer Research Society (13). An effort was taken to section all specimens at 2 mm interval and centered on the lesion with the most profound invasion's closes margin location. Only slides that had been stained by hematoxylin-eosin were utilized in the general assessment. The magnitude and invasion depth of the tumor, lymphatic as well as vascular movement, and the tumor's contribution at the vertical and lateral margins were examined histologically.

### Valuation of Efficacy of Resection

En bloc resection is termed as removing a tumor is a single-piece absent of potential disintegration. The entire resection for an en bloc resected tumor is regarded as all the vertical and lateral margins having no tumors during the histological examination. Tumors that histologically had positive resection margins or multiple fragments were regarded as partial resection. When the lesion is resected en bloc, the following conditions: (i) predominantly differentiated type, pT1a,UL0, HM0 VM0, Ly0, V0, regardless of size; (ii) long diameter ≤ 2 cm, predominantly undifferentiated type, pT1a , UL0, HM0, VM0, Ly0, V0; or (iii) long diameter ≤3 cm, predominantly differentiated type, pT1a, UL1,HM0, VM0, Ly0, and V0 are considered for endoscopic curability A (eCuraA); When the lesion is resected en bloc, is ≤3 cm in long diameter, predominantly of the differentiated type, and satisfies the following criteria:pT1b1 (SM1) (within <500 mm from the muscularis mucosae), HM0, VM0, Ly0, and V0, it is considered endoscopic curability B (eCuraB); When a lesion meets neither of the above-mentioned eCuraA and B conditions, it is considered eCuraC, which corresponds to the concept of non-curative resection. When eCuraC lesions are differentiated-type lesionsand fulfill other criteria to be classified into either eCuraA or eCuraB but was either not resected en bloc or had positive HM, they are considered eCuraC-1. All other eCuraC lesions are considered eCuraC-2 (14).

### A Predictive Framework for the Non-curative Resection of ESD

Only the threat conditions that demonstrated numerical worth were adopted in the development of a predictive framework or model. We incorporated risk factors (the results of multivariate log-binomial regression) and potential clinical indicators into the model to optimize its predictive power. According to the results of multivariate log-binomial regression, a nomogram was drawn. By drawing a calibration chart, the ROC curve was executed to obtain the area under the curve (AUC). The C-index was calculated to evaluate the predictive effect of the nomogram (15).

### Statistical Analysis

The presentation of the continuous variables is as mean ± standard deviation. The definite variables are shown as figures with percentages. Univariable analysis was performed to categorize the aspects related to non-curative resection of ESD/EMR utilizing a chi-square test. Univariate analysis and statistically significant differences (*P* < 0.05) were further included in the multivariate regression analysis. Then, multivariate regression analysis was used to select independent influence factors, and nomograms were built based mainly on these results. The area under the curve (AUC) for validation was applied to evaluate the accuracy of the nomograms. We performed calibration for the established nomograms and applied 1,000 repetitions of bootstrap sample corrections to internally validate the nomograms. The team only included the variables of *P*-values lower than 0.05 that were regarded as statistically meaningful. Calculations were undertaken using SPSS software using the latest version, version 24.0 (SPSS, Chicago, Ill, USA). Nomogram drawn was performed using R Software 4.0.4 (www.r-project.org). Package “rms” was used for nomogram building.

## Results

### Baseline Features of Patients

**Figure 1:**
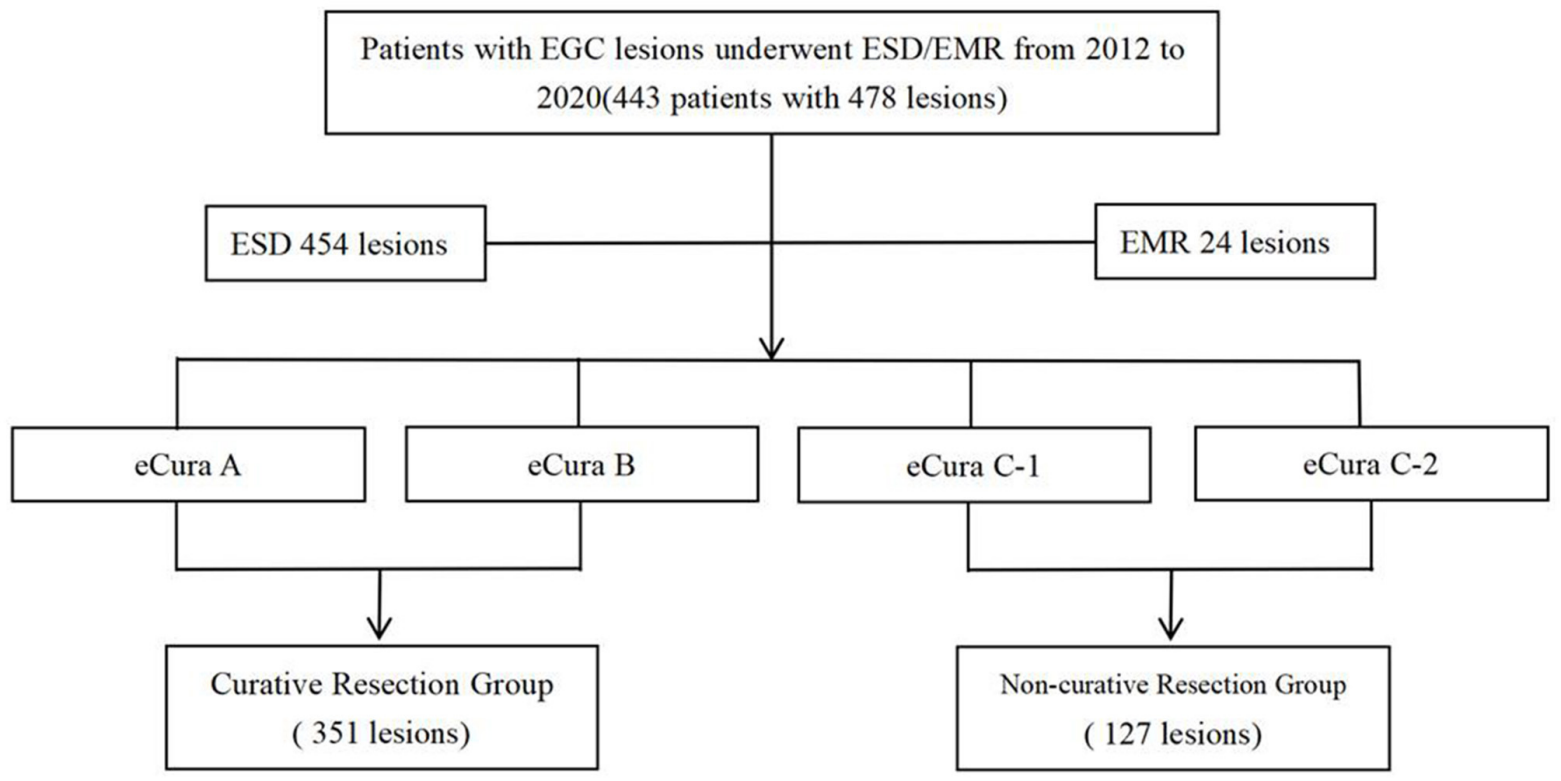
Study design. A total of 443 patients, including the curative resection group and the non-curative resection group, were reviewed retrospectively.

Four hundred forty-three patients with four hundred seventy-eight lesions were suffering from EGC. Among them, there were 344 males and 134 females, with an average age of 63.28 years. There were 127 cases of non-curative resection of early gastric cancer and 351 cases of curative resection. In 454 cases of en bloc resection, the en bloc resection rate was 95.0%, and the non-curative resection rate of the total included patients was 26.6% (127/478) (Figure 1).

The characteristics and clinicopathology of the patient are highlighted in Table 1. Patients older than 60 years are more prone to developing non-curable resection (*P* = 0.007). The group composed of non-curative resection had larger tumors than groups composed of curative resection (1.77 ± 1.05 cm vs. 2.40 ± 1.85 cm; *P* < 0.001). The tumors located on the stomach's upper body part were more susceptible to occur in patients from the group of non-curative resection relative to the group for curative resection (*P* < 0.001). Ulcers occurred in different degrees between the two groups. In the non-curative resection group, 13 cases of undifferentiated cancer were diagnosed before operation, accounting for 10.7%, and 3 cases of undifferentiated cancer were diagnosed before curative resection, accounting for 0.8%. The difference was statistically significant (*P* < 0.001). Additionally, postoperative pathological diagnosis showed that 27 cases of undifferentiated cancer were non-curable resection, accounting for 21.3%, while curative resection was only 12 cases, accounting for 3.4% (*P* < 0.001); in terms of the depth of tumor invasion, in the non-curative resection group, there were 45 cases of submucosal tumors, accounting for 35.5%. In the curative resection group, there were 13 cases of submucosal tumors, accounting for only 3.7%. The difference was statistically significant (*P* < 0.001; Table 1).

**Table 1:** Patient's clinicopathologic characteristics (curative resection group vs. non-curative resection group).

**Variable**	**Curative resection (*n* = 351)**	**Non-curative resection (*n* = 127)**	***p*-value**
Age, year, mean ± SD	62.70 ± 9.34	64.87 ± 10.32	0.030
≥60, year	131 (37.3)	30 (23.6)	0.007
Male, *n* (%)	254 (72.4)	90 (70.9)	0.836
Smoking history, *n* (%)	142 (40.5)	62 (48.8)	0.126
Alcohol history, *n* (%)	109 (31.1)	44 (34.6)	0.527
Family history of tumor, *n* (%)	96 (27.4)	46 (36.2)	0.078
**Co-morbidity disease**			
Hypertension, *n* (%)	129 (36.8)	46 (36.2)	>0.999
Hyperlipidemia, *n* (%)	45 (12.8)	23 (18.1)	0.189
Diabetes mellitus, *n* (%)	53 (15.1)	24 (18.9)	0.392
Cardiovascular disease, *n* (%)	68 (19.4)	30 (23.6)	0.374
**Pre-procedure diagnosis**			< 0.001
Adenoma or atypical cells, *n* (%)	87(24.4)	22(18.0)	
Differentiated, *n* (%)	266(74.7)	87(71.3)	
Undiffrentiated, *n* (%)	3(0.8)	13(10.7)	
**Post-procedure diagnosis**			< 0.001
Differentiated, *n* (%)	339(96.6)	100(78.7)	
Undifferentiated, *n* (%)	12 (3.4)	27 (21.3)	
**Tumor location, long axis**, ***n*** **(%)**			< 0.001
Lower	87 (24.8)	49 (38.6)	
Middle	43 (12.3)	25 (19.7)	
Upper	221 (63.0)	53 (41.7)	
**Tumor location, short axis**, ***n*** **(%)**			0.475
Lesser curvature	102 (29.1)	40 (31.5)	
Greater curvature	55 (15.7)	24 (18.9)	
Posterior wall	120 (34.2)	34 (26.8)	
Anterior wall	74 (21.1)	29 (22.8)	
**Gross type**, ***n*** **(%)**			0.343
Elevated	189 (53.8)	59 (46.5)	
Flat	63 (17.9)	25 (19.7)	
Depressed	99 (28.2)	43 (33.9)	
Tumor size, cm	1.77 ± 1.05	2.40 ± 1.85	< 0.001
**Endoscopic findings**, ***n*** **(%)**			
Ulcer	42 (12.0)	34 (26.8)	< 0.001
Remarkable redness	152 (43.3)	42 (33.1)	0.057
Central depression	90 (25.3)	38 (31.1)	0.098
Interruption or smooth tapering of fold	4 (1.1)	6 (4.7)	0.040
White fur	24 (6.8)	19 (15.0)	0.010
Nodularity	18 (5.1)	10 (7.9)	0.364
**Depth of tumor**, ***n*** **(%)**			< 0.001
Mucosal lesion	338 (96.3)	82 (64.6)	
Submucosal lesion	13 (3.7)	45 (35.5)	
**Complication**, ***n*** **(%)**			
Bleeding during procedure	7 (2.0)	4 (31.0)	0.690
Bleeding after procedure	18 (5.1)	9 (7.1)	0.552
**Hp infection**, ***n*** **(%)**
Negative	188 (52.8)	46 (37.7)	0.004
Positive	26 (7.3)	6 (4.9)	
Not tested	142(39.9)	70(57.4)	
**Treatment**			< 0.001
ESD	343 (75.4)	112 (24.6)	
EMR	8 (34.7)	15 (65.2)	
Piecemeal resection	0 (0)	24 (18.9)	< 0.001

*SD, standard deviation; ESD, endoscopic submucosal dissection; EMR, endoscopic mucosal resection; Hp, Helicobacter pylori*.

### Risk Factors for Non-curative Resection of ESD

The results of univariate analysis showed that patients ≥60 years of age were more likely to have non-curative resection (OR = 1.890; *P* = 0.007); patients with a family history of tumors were more likely to have non-curative resection (OR = 1.485; *P* = 0.078). The diameter of the tumor in the non-curative resection group was larger than that in the curative resection group (OR = 1.054; *P* < 0.001); compared with the curative resection group, the upper half of the stomach tumor in the non-curative resection group was more common (OR = 2.385; *P* < 0.001). For the endoscopic manifestations of the lesions, the ulcers between the two groups were different (OR = 2.836; *P* < 0.001); in addition, whether the non-curative resection group and the curative resection group were en bloc resection (OR = 1.231; *P* < 0.001), Hp infection (OR = 1.088; *P* = 0.001), and treatment methods (OR = 9.768; *P* = 0.762). The histological undifferentiated tumors in the non-curable resection group were more common than those in the curative resection group (27 vs. 12, OR = 8.147; *P* < 0.001).

In the multivariate analysis, older age (>60 years; OR = 2.558; 95% CI = 1.280–5.111), a large tumor size (>30 mm) (OR = 3.952; 95% CI = 1.397–11.184), the treatment modality is EMR (OR = 4.581; 95% CI = 1.526–13.748), piecemeal resection (OR = 63.021; 95%CI = 12.270–323.687), with submucosal infiltration (OR = 2.496; 95% CI = 1.727–3.607), and undifferentiated tumor histology (OR = 4.917; 95% CI = 1.591–15.195) were associated with non-curative resection (Table 2).

**Table 2:** Associated factors with non-curative resection of ESD/EMR.

**Variable**	**Multivariate analysis**			***P-*value**
	**OR**	**95% CI**		
**Age**				
<60 year	1			
≥60 year	2.558	1.280	5.111	0.008
**Gender**				
Female	1			
Male	1.581	0.845	2.960	0.152
**Post-procedure diagnosis**				
Differentiated	1			
Undifferentiated	4.917	1.591	15.195	0.006
**Tumor location, long axis**				
Lower	1			
Middle	1.016	0.403	2.558	0.974
Upper	0.987	0.486	2.003	0.971
**Tumor size**				
<3 cm				
≥3 cm	3.952	1.397	11.184	0.010
**Ulcer**				
N	1			
Y	1.664	0.775	3.573	0.191
**Interruption or smooth tapering of fold**				
N	1			
Y	2.557	0.220	29.782	0.454
**Depth of tumor**				
Mucosal lesion	1			
Submucosal lesion	2.49	1.73	3.61	<0.001
**En bloc resection**				
Y	1			
N	63.021	12.270	323.687	<0.001
**HP infection**				
Negative	1			
Positive	1.604	0.835	3.083	0.156
Not tested	0.664	0.153	2.890	0.585
**Resection method**				
ESD	1			
EMR	4.581	1.526	13.748	0.007

*OR, odds ratio; CI, confidence interval; ESD, endoscopic submucosal dissection; EMR, endoscopic mucosal resection. Hp, Helicobacter pylori*.

### The Prediction Model Grounded on Independent Risk Factors

**Figure 2:**
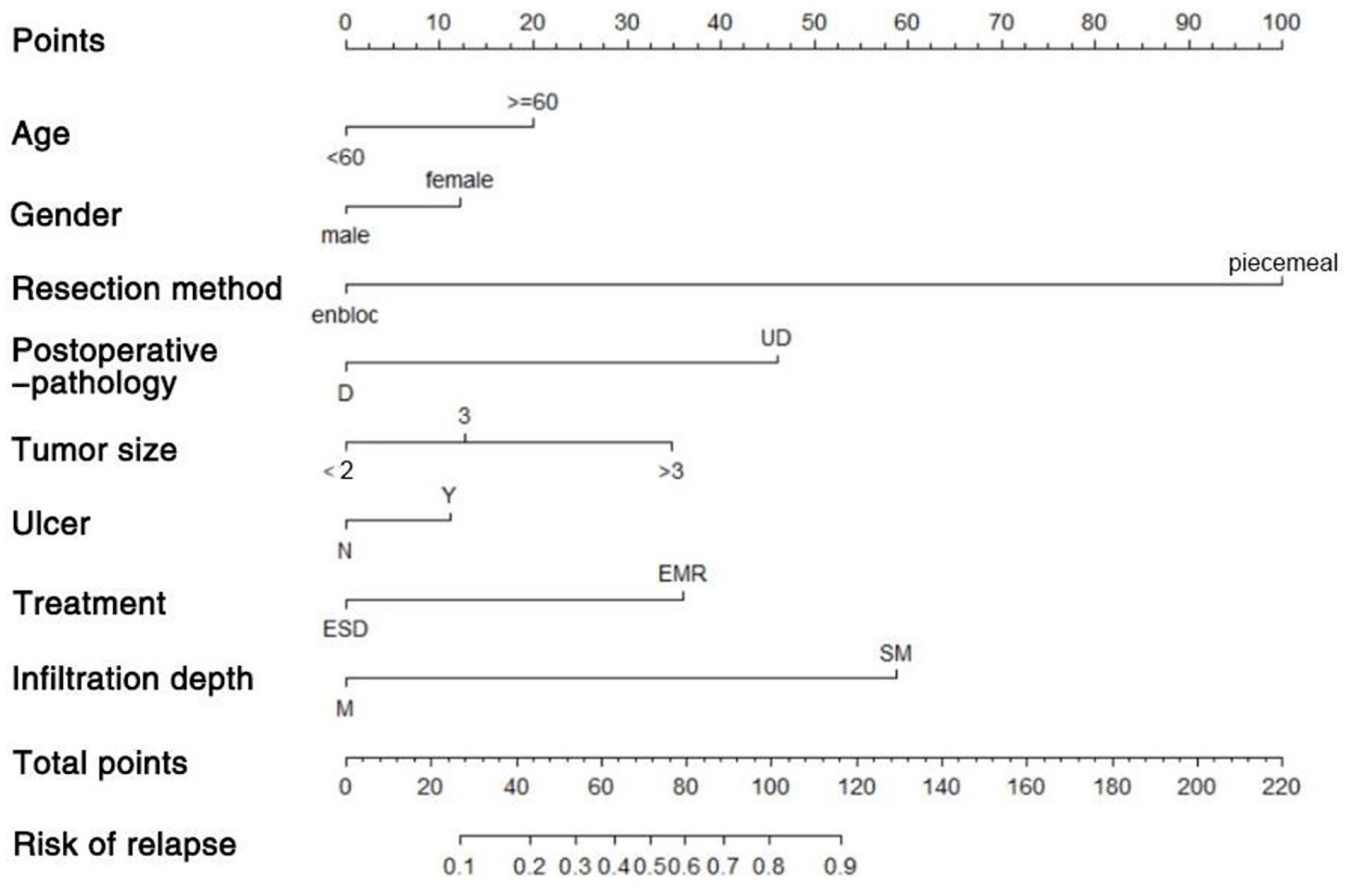
Predictive nomogram for the EGCs undertaking ESD/EMR potential to develop non-curative resection.

**Figure 3:**
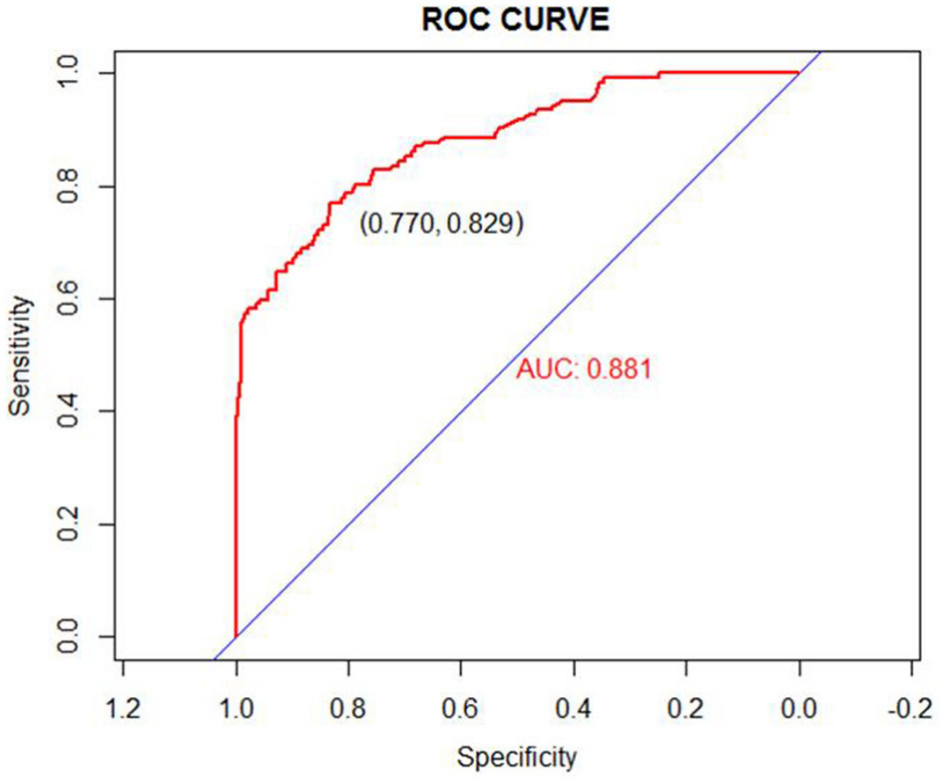
ROC curve for our prediction nomogram model. Area under the ROC curve = 0.881 (ROC, receiver operating curve).

**Figure 4:**
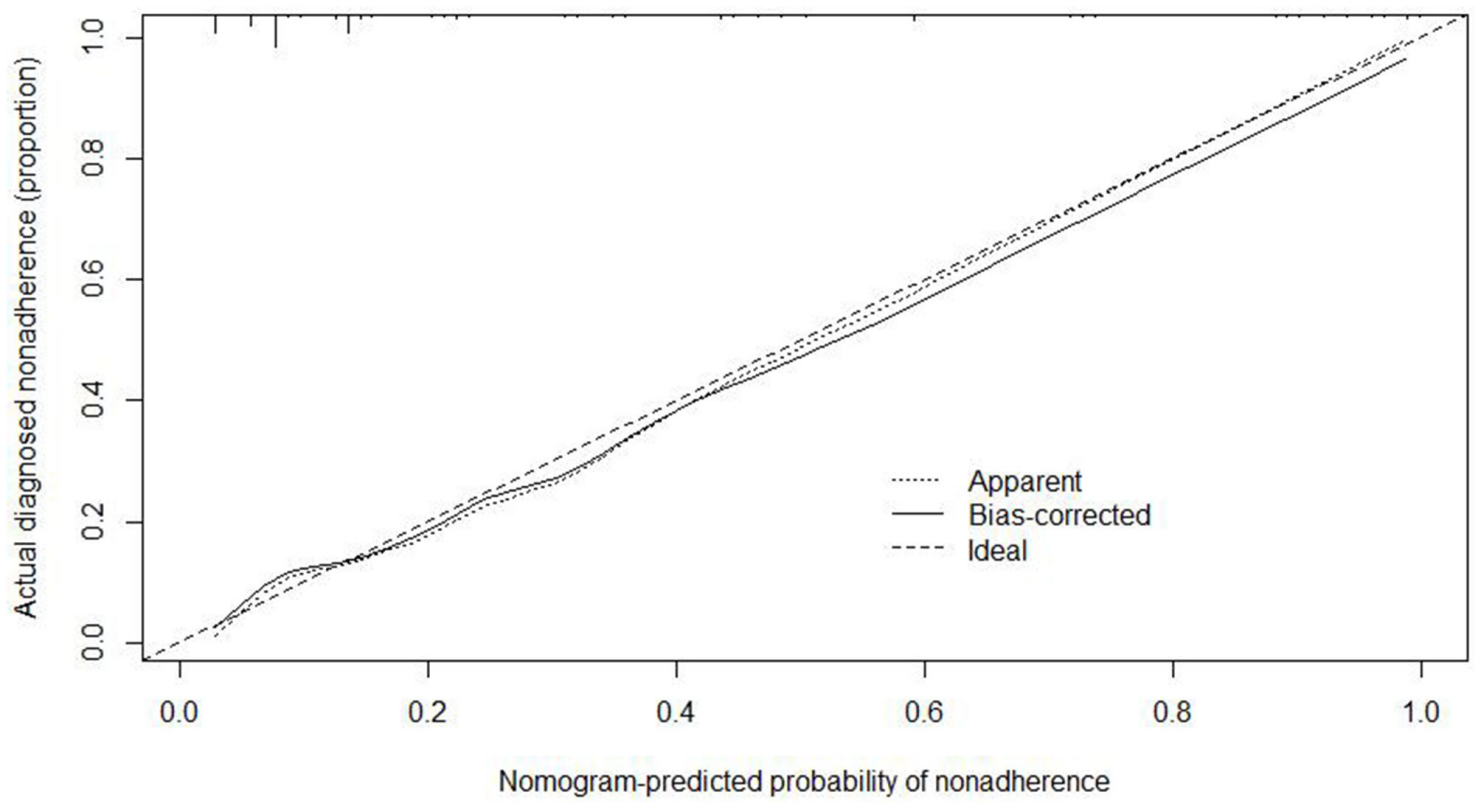
Calibration curve of this nomogram model.

We used the independent risk factors (age, gender, resection method, postoperative pathology, tumor size, and depth of tumor corresponding to infiltration depth in the Figure 2) to develop a predictive nomogram (Figure 2) for the EGCs undertaking ESD/EMR potential to develop non-curative resection. Two additional factors (gender and ulcer) were also included, considering their corresponding OR value in univariate analysis. For each patient, points were assigned for each of these demographic and medical factors (age, gender, resection method, postoperative pathology, tumor size, ulcer, and depth), then a total score and a corresponding prediction of the probability of non-curative resection were calculated from the nomogram. An ROC curve was drawn to estimate the predictive accuracy of the nomogram, and the AUC (95% CI) was 0.881 (Figure 3). A calibration curve generated by 1,000 repetitions of bootstrap sample corrections is illustrated in Figure 4.

## Discussion

With the development and broad application of the current early screening technology and minimally invasive endoscopic technology, more and more early gastric cancers are detected and effectively treated (16). ER (Endoscopic Resection), consisting of endoscopic submucosal dissection (ESD), and mucosal resection (EMR), is broadly acknowledged as a marginally invasive procedure for EGC. ER leads to a decent lasting result, including a 97.5% 5-year endurance rate, quality of life, and less morbidity than surgical treatment (3). EMR is the technique to treat flat and raised lesions (early gastrointestinal cancer, flat adenoma) through endoscopic measures (injection and suction) to separate the lesion from the lamina propria, then trapped or cut. ESD is an endoscopic submucosal injection and then using a special high-frequency electric knife to peel off the lesion's mucosa to realize the objective of treatment. Due to the limitations of EMR, endoscopic submucosal dissection has turned out to be the leading procedure in the cure of EGC.

Nevertheless, endoscopic resection is not a definite method in the treatment of EGC. Clinical cases of non-curative resection are common, and endoscopic resection for EGC is often caused by insufficient preoperative evaluation, and lack of experience of the surgeon leads to non-curative resection. In certain instances, patients suspected to lie within the criteria prior to surgery can be ascertained as cases over the extended signs based on the final histopathologic result.

Different threat aspects linked to non-curative resection of ESD in EGC or lymph node metastasis are printed within existing studies (17, 18). However, research directed at various endoscopic findings that include atrophy, fold shape, or exudate is rare. Previous studies only focused on individual risk factors and did not consider these risk factors comprehensively. We wanted to find useful risk clinical factors and establish a predictive model that can be used before deciding whether to perform ESD/EMR.

Our study determined numerous endoscopic results that included the size of the tumor, patient age, location of the tumor, presence of ulcers, and the indistinguishable type of histology associated with a higher threat level for non-curative resection. The team formulated a predictive model for scoring consisting of these aspects. The presence of ulcers was identified as a leading prognostic factor associated with EGC's curability with endoscopic submucosal dissection (19, 20). Consistent with prior research, the evidence of ulcers was determined to be linked with non-curative resection in our research. For lesions with ulcer formation, submucosal adhesions are often found during the operation, which makes the lifting of the submucosal injection poor, increases the difficulty and risk of the operation, and may affect the curative resection rate.

Undifferentiated/poorly differentiated histological types are also related to non-curative resection after endoscopic resection. Undifferentiated histological types have been identified as significant threat influences for non-curative resection by many studies (10, 18). However, with the development of endoscopy, more and more studies believe that ESD is safe and effective for treating undifferentiated histological types (21, 22). Therefore, the latest Japanese guidelines suggest that undifferentiated EGC (≤2 cm) formed by ulcers can be treated with ESD (14). However, it should be carefully considered when determining whether or not to perform an endoscopic submucosal dissection on people with undifferentiated EGC. Especially in indistinguishable EGC, for the limitation of the lesion's size, we can see that there is a difference in the measurement of the diameter of the lesion before and after endoscopic resection. Hence, the likelihood of non-curative resection following ESD has to be taken into account for patients with undifferentiated histology.

Nomograms as risk estimators have shown promising potential in clinical trial design and interpretation and have been widely adopted in prognostic models. In this study, we established a nomogram-based method to select the high-risk patients that have undergone EMR or ESD to non-curative resection based on different risk factors. The threat of non-curative resection among patients that have undergone EMR or ESD can be stratified using the team's predictive framework. Compared with the prediction model by Hyeong Seok Nam (23), which used multivariate regression analysis to derive the risk factors for non-curative resection, and calculate the number of these risk factors and a high number of risk factors were associated with an increased frequency of non-curative endoscopic resection. Our nomogram used the regression coefficients and receiver operating characteristic (ROC) curve analysis and calibration to present the predictive accuracy of the nomogram. Kim EH (24), etc., built a risk scoring model assigned for these variables based on the beta-coefficient as follows: tumor size (≥20 mm); tumor location in the upper body of the stomach; ulcer; fusion of gastric folds; absence of mucosal nodularity; spontaneous bleeding and undifferentiated histology. The area under the receiver-operating characteristic curve is 0.7004. Our risk nomogram showed better discriminated performance in internal validation (bootstrap-corrected area under the receiver-operating characteristic curve, 0.881; *P* < 0.001). Besides, the nomogram chart is more intuitive, and because the coefficients in the multiple regression are used accurately in the establishment process, the prediction probability will not be biased due to the scoring. To our knowledge, this is the first study providing a nomogram to predict NCR risk undergone EMR or ESD.

There are also some limitations in our study. First, the nomogram was based on a retrospective single-center dataset, which would weaken the confidence of our risk prediction model and shrink its application range. Second, the team only carried out a validation exercise to demonstrate the validity of the team's model. External validation was crucial in demonstrating the model's precision and may cause statistical analysis bias after elimination. To establish a perfect predictive model, the threat conditions of non-curative resection after endoscopic resection for EGC still require further multi-center, large-sample clinical studies to provide more evidence.

The aim of this research is to develop a predictive framework for non-curative resection utilizing viable clinical factors. Hence, our extrapolative framework will offer valuable data on decision-making process concerning early gastric cancer treatment before EMR or ESD.

## Data Availability Statement

The original contributions presented in the study are included in the article. Further inquiries can be directed to the corresponding author.

## Ethics Statement

The studies involving human participants were reviewed and approved by Capital Medical University affiliated Beijing Friendship Hospital. The patients/participants provided their written informed consent to participate in this study. Written informed consent was obtained from the individual(s) for the publication of any potentially identifiable images or data included in this article.

## Author Contributions

SZha and XS: conceptualization. QZ: methodology, resources, and data curation. XM: software, writing—original draft preparation, and formal analysis. XM, QZ, and SZhu: validation. SZhu: investigation. XS: writing—review and editing. SZha: visualization, supervision, and project administration. All authors have read and agreed to the published version of the manuscript.

## Conflict of Interest

The authors declare that the research was conducted in the absence of any commercial or financial relationships that could be construed as a potential conflict of interest.

## Abbreviations

ESD: endoscopic submucosal dissection
EGC: early gastric cancer
EMR: endoscopic mucosal resection
APC: argon plasma coagulation
SD: standard deviation
OR: odds ratio
CI: confidence interval.
